# The negative health effects of hostile environment policies on migrants: A cross-sectional service evaluation of humanitarian healthcare provision in the UK

**DOI:** 10.12688/wellcomeopenres.15358.1

**Published:** 2019-07-22

**Authors:** Sophie J. Weller, Liam J. Crosby, Eleanor R. Turnbull, Rachel Burns, Anna Miller, Lucy Jones, Robert W. Aldridge

**Affiliations:** 1 Academic Foundation Programme, North Central and East London Foundation School, Health Education England, Stewart House, 32 Russell Square, London, WC1B 5DN, UK; 2 School of Public Health, St Mary's Hospital, Imperial College London, Praed St, Paddington, London, W2 1NY, UK; 3 Centre for Public Health Data Science, Institute of Health Informatics, University College London, London, NW1 2DA, UK; 4 Doctors of the World UK, 29th Floor, One Canada Square, London, E14 5AA, UK

**Keywords:** refugees, migrants, undocumented, asylum, UK, health, barrier, hostile environment

## Abstract

**Background: **Recent UK ‘hostile environment’ immigration policies, including obligatory charging and sharing of confidential data between NHS Digital and the Home Office, have created an atmosphere of fear and exposed already highly marginalised and vulnerable groups to significant health risks by increasing barriers to accessing NHS care.

**Methods:**  This is a cross-sectional observational study of patients accessing healthcare at Doctors of the World (DOTW) in the UK. DOTW is a humanitarian organisation, providing care to those excluded from NHS healthcare. We aimed to describe population characteristics of individuals using DOTW services and identify groups at greatest risk of facing ‘hostile environment’-related barriers to NHS care, specifically being denied healthcare or fear of arrest.

**Results:** A total of 1474 adults were seen in 2016. Nearly all were non-EU/EEA nationals (97.8%; 1441/1474), living in poverty (68.6%; 1011/1474). DOTW saw a large number of undocumented migrants (57.1%; 841/1474) and asylum seekers (18.2%; 268/1474). 10.2% (151/1474) of adults seen had been denied NHS healthcare and 7.7% (114/1474) were afraid to access NHS services. Asylum seeker status was associated with the highest risk (adjusted odds ratio (OR): 2.48; 95% confidence interval (CI): 1.48-4.14) of being denied NHS healthcare and being undocumented was associated with the highest risk of fearing arrest (adjusted OR: 3.03; 95% CI: 1.70-5.40).

**Conclusions: **Our findings make visible the multiple and intersecting vulnerabilities of individuals forced to seek care outside of the NHS, underlining the public health imperative for the government to urgently withdraw its ‘hostile environment’ policies and address their negative health impacts.

## Introduction

There are an estimated 258 million international migrants worldwide, of which 25.9 million are refugees and asylum seekers ^1^. The UN Secretary General described global migration as “one of the most urgent and profound tests of international cooperation in our time” ^2^. Experts in the field have called for the active promotion and protection of the health of migrants ^3^, while the Academy of Royal Colleges of Medicine has called publicly for the rescindment of policies that hinder migrants’ access to healthcare ^4^. The right to health for all is one of a set of internationally agreed human rights standards, detailed under the International Covenant on Economic, Social and Cultural Rights ^5^.

In 2012, in stark contrast to this public health imperative and international human rights laws, the recent UK Prime Minister and then Home Secretary, Theresa May stated her aim “ *was to create here in Britain a really hostile environment for illegal migration”* by denying individuals without legal documentation everything they needed to survive, including healthcare ^6^. The UK’s Immigration Acts of 2014 and 2016 extended ‘hostile environment’ immigration policies into far-reaching aspects of public life. This led to mandatory upfront charges to ‘non-ordinary residents’ for National Health System (NHS) secondary care, including undocumented migrants and those denied asylum. Some migrants are exempt from charges; however, a lack of understanding of the rules and difficulties in proving exemption means people are wrongly denied or charged for care ^7^.

An extension of the hostile environment policies was highlighted by the discovery of a much-criticised Memorandum of Understanding between NHS Digital and the Home Office ^8, 9^ which facilitated the sharing of confidential non-clinical information about patients for immigration enforcement purposes. Though this MOU was withdrawn in 2018, patient information collected by NHS trusts is still shared with the Home Office ^10^ and these extreme measures have extended an environment of mistrust, increasing the direct and indirect barriers that many migrants face in accessing healthcare: many fear arrest, are incorrectly denied access, or are unable to afford care ^11^.

Migrants who are unable to access NHS services and in desperate need of healthcare seek assistance at the clinics of the international humanitarian organisation, Doctors of the World (DOTW). These clinics provide walk-in, free primary care and health advice, consultation for broader social problems, and advocacy to enable people to register with NHS GPs. Importantly, awareness of these clinics is often through word of mouth and via trusted contacts and as such these clinics provide services to a highly marginalised group of individuals who are unable or too fearful to access healthcare elsewhere. Attendance at these clinics is evidence of exclusion from mainstream healthcare provision.

Doctors of the World’s recent Médecins du Monde Observatory Report ^12^, based on data from 43,286 people attending clinics run by DOTW and partner NGOs across Europe in 2016, found 55.2% of attendees had no access to healthcare coverage at all, with 18.3% accessing emergency care only. Individuals sought medical attention for both acute and chronic conditions and over half of pregnant women were not accessing antenatal services. The report described a vulnerable and highly excluded population where social isolation was common. Most notably the highest number of responses reporting fear of arrest and denied access to healthcare was in the UK.

There is a need to identify the characteristics and healthcare needs of this highly marginalised group at the country-specific level, in order to evaluate and improve the services provided by DOTW, hold national policy makers to account, inform future policy and advocate for meaningful public health interventions. This study contributes to this goal by looking specifically at data collected in the United Kingdom, during the preparation of the Médecins du Monde Observatory Report, and considers these data within the context of prevailing UK government policy. The objectives of the study are to evaluate the characteristics of the population using DOTW services, i.e. those excluded from NHS services, and identify those groups at greatest risk of facing ‘hostile environment’-related barriers to NHS care.

## Methods

### Study design

We conducted a cross-sectional humanitarian health service evaluation using data collected during all consultations at DOTW clinics in Bethnal Green and outreach locations in London and Brighton, from 1st January to 31st December 2016. As this was a service evaluation all adults (>18 years old) attending the clinics were eligible for inclusion; those aged <18 years were excluded. Attendees at DOTW clinics underwent two consultations, one “social” and one “medical”. The “social” consultation was conducted by casework volunteers and covered issues such as housing, immigration, income and health access. “Medical” consultations were conducted by doctors and focused on current health, medical history, pregnancy and vaccination. All information was entered into a database during the consultation. For this analysis we focus solely on data collected using the “Social” form (available as *Extended data* ^13^).

Age was grouped into age-band variables and EU national status was assigned based on self-reported nationality (where dual nationality was reported and one nationality was in the EU, EU nationality was recorded). Income was coded as ‘above’ or ‘below’ a poverty threshold of £1050/month. As income was missing for many (n=349) participants, these were assigned as ‘income data missing’ to allow them to be retained within regression models. Immigration status was coded as either ‘asylum seeker’ (defined as a person who seeks safety from persecution or serious harm in a country other than his or her own and awaits a decision on the application for refugee status under relevant international and national instruments), ‘undocumented’ (defined as a person who does not have the necessary documentation to enter or remain legally in a country) or ‘permissions to reside (other than asylum)’. The ‘permissions to reside (other than asylum)’ immigration status group consisted of individuals whose status was categorised as one of the following: has valid residency permit and permit end date; tourist; student; short stay visa; work visa; humanitarian protection/discretionary leave; permit to stay in another EU country and here for <3 months; specific situations giving a right to stay (specific per country): under process for sorting out papers; or unknown.

Where an individual attended a DOTW clinic multiple times, we selected data from the record with the most complete data for that individual, with a preference for the first attendance if data were equally complete.

### Outcomes

Descriptive analysis was used to examine the characteristics of those using DOTW services. We interrogated data on age, gender, nationality, income, housing, parental status, immigration status and barriers in access to NHS healthcare. In further analysis we sought to investigate which population characteristics were associated with facing hostile-environment-related barriers to NHS care. The two hostile-environment-related outcomes that we investigated were whether people disclosed (a) being denied health coverage or (b) fear of being reported or arrested, as a reason for not accessing NHS care within the past 12 months. As each individual could give multiple reasons for not accessing NHS care, we created two separate variables, one for each of the responses noted above.

### Statistical methods

Descriptive results are presented using counts and percentages. Data are suppressed where cell counts would be less than 10, or where column or row totals could be used to deduce suppressed cell counts. We conducted multivariable logistic regression analyses to investigate the effect of explanatory variables on the likelihood of experiencing each of the outcome measures, respectively, having adjusted for other explanatory variables. We conducted a series of sensitivity analyses to explore the effects of excluding missing data on the final logistic regression models presented in our analyses. Data were stored and analysed using Stata version 15 at University College London (UCL).

### Ethics

Prior to undertaking this analysis, this project was reviewed by the joint chairs of UCL’s Research Ethics Committee, who deemed it exempt from requiring ethics clearance on the basis that it constituted a service evaluation aiming to improve understanding of demand for and access to DOTW services and who deemed that participant consent was not required. DOTW provided anonymised data for this analysis.

## Results

### Study population demographics

**Figure 1.**
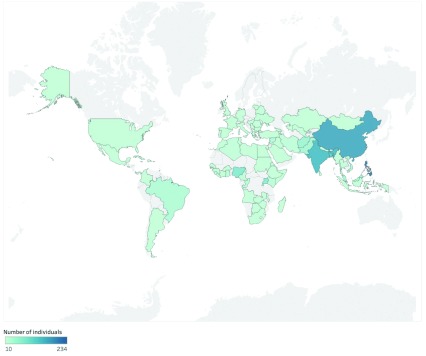
Global map of nationalities of individuals.

In total, 1543 individuals were seen at DOTW clinics in the UK in 2016. After removal of individuals aged under 18, we were left with 1474 individuals for analysis. Table 1 shows the study population demographic characteristics. Notably, attendees were of fairly equal gender (52.0% female; 767/1474); the majority were aged between 18–34 years (43.0%; 634/1474) and of non-EU/EEA nationality (97.8%; 1441/1474). 478 (32.4%; 478/1474) individuals lived in unstable housing, 1011 (68.5%; 1011/1474) were living under the poverty threshold and 655 (44.4%; 655/1474) had dependent children. In terms of immigration status, 268 individuals (18.1%; 268/1474) were asylum seekers whilst 841 (57.1%; 841/1474) were undocumented. Figure 1 shows the wide global distribution of individuals seeking care at DOTW clinics. The top three nationalities for individuals accessing the DOTW clinic were Filipinos (15.9%; 234/1474); Chinese (11.5%; 169/1474) and Indian (10.0%; 147/1474).

**Table 1.** Baseline demographic characteristics of individuals. Note: to avoid deductive disclosure, results with less than 10 records have been suppressed.

Variable		Reason for not using NHS services	
	All	Denied Healthcare *	Fear of Arrest *
All	1474 (100)	151 (100)	114 (100)
Gender			
Female	767 (52.0)	67 (44.4)	67 (58.8)
Male	707 (48.0)	84 (55.6)	47 (41.2)
Age of individual at consultation			
18–34	634 (43.0)	60 (39.7)	43 (37.7)
35–44	429 (29.1)	47 (31.1)	38 (33.3)
45+	393 (26.7)	40-50 (-)	30-40 (-)
Not known	18 (1.2)	<10 (-)	<10 (-)
Has dependent children			
Yes	655 (44.4)	59 (39.1)	51 (44.7)
No	819 (55.6)	92 (60.9)	63 (55.3)
Housing situation			
Stable housing	924 (62.7)	107 (70.9)	72 (63.2)
Unstable housing	478 (32.4)	40-50 (-)	30-49 (-)
Not known	72 (4.9)	<10 (-)	<10 (-)
Income level in last 3 months			
Under poverty threshold	1011 (68.6)	109 (72.2)	83 (72.8)
Over poverty threshold	151 (10.2)	16 (10.6)	14 (12.3)
Not known	312 (21.2)	26 (17.2)	17 (14.9)
EU National			
Yes	30-40 ()	<10 (-)	- (-)
No	1441 (97.8)	140-151 (-)	114 (100)
Not known	<10 ()	<10 (-)	- (-)
Immigration status			
Asylum seeker	268 (18.2)	47 (31.1)	<10 (-)
Undocumented	841 (57.1)	76 (50.3)	93 (81.6)
Permissions to reside (other than asylum)	365 (24.8)	28 (18.5)	10-20 (-)

*n (% of column total)

### Barriers to accessing NHS healthcare

**Figure 2.**
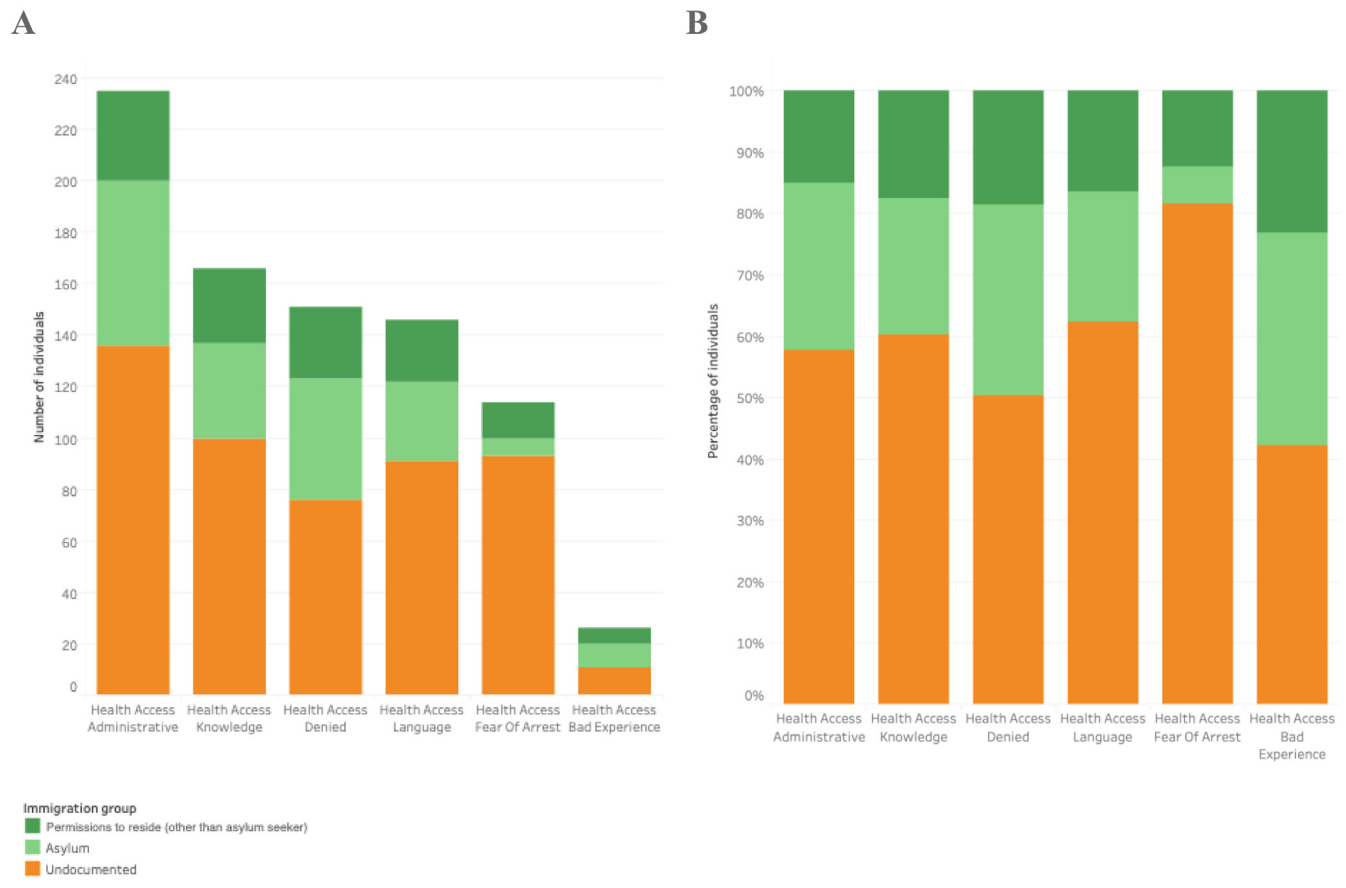
Health barriers by immigration sub-group. ( **A**) Total number of individuals reporting health access barriers by immigration sub-group. ( **B**) Percentage of individuals reporting health access barriers by immigration sub-group.

 We examined barriers to accessing NHS healthcare in this population ( Figure 2), with individuals reporting one or more barriers where applicable. Administrative barriers were reported at the highest levels (15.9%; 235/1474), followed by a lack of knowledge on how to access NHS care (11.3%; 166/1474), previously having been denied access to care (10.2%; 151/1474), and language barriers (9.9%; 146/1474). We analysed the healthcare access barriers by immigration status. For undocumented migrants, fear of arrest (81.6%; 93/114) was the healthcare access barrier they were at greatest risk of experiencing compared to other immigration status groups ( Figure 2B). For asylum seekers, a previous bad experience was the healthcare access barrier they were at greatest risk of experiencing (however, as the total number was <10 this was not reported) , compared to other immigration status groups, followed by denial of access to healthcare (31.1%; 47/151) and administrative reasons (27.2%; 64/235).

Overall, 151 individuals (10.2%; 151/1474) had been denied NHS healthcare before seeking help at a DOTW clinic. Males (55.6%; 84/151), people who had claimed asylum (31.1%; 47/151), and those with no dependent children (60.9%; 92/151) were more likely to have been denied NHS care in crude analysis (i.e. before adjusting for other factors). Table 2 presents the results of our multivariable logistic regression examining the risk factors for denial of access to healthcare and fear of arrest upon seeking healthcare. People who had made an asylum claim were more than two times likely to have been denied NHS care compared to those with permissions to reside (adjusted odds ratio (OR): 2.48; 95% confidence interval (CI): 1.48-4.14) after adjusting for age, gender and income level.

**Table 2.** Logistic regression results of risk factors for denial of access to healthcare and fear of arrest.

Variable	Denied Healthcare		Fear of Arrest	
	OR (95%CI)	p-value	OR (95%CI; )	p-value
Gender				
Female	1.0		1.0	
Male	1.28 (0.91-1.81)	0.5899	0.79 (0.53-1.17)	0.2381
Age of individual at consultation				
18–34	1.0		1.0	
35–44	1.21 (0.80-1.81)		1.26 (0.80-2.00)	
45+	1.28 (0.84-1.94)	0.4619	1.15 (0.71-1.86)	0.5989
Income level in last 3 months				
Under poverty threshold	1.0		1.0	
Over poverty threshold	1.19 (0.68-2.1)		1.08 (0.59-1.98)	
Not known	0.85 (0.54-1.34)	0.5899	0.66 (0.38-1.14)	0.2689
Immigration status				
Permissions to reside (other than asylum seeker)	1.0		1.0	
Asylum seeker	2.48 (1.48-4.14)		0.69 (0.27-1.75)	
Undocumented	1.16 (0.73-1.83)	0.0004	3.03 (1.70-5.40)	<0.0001

*OR = odds ratio; p-value based upon likelihood ratio test; all models adjusted for gender, age; income and immigration status.

In total, 114 people (7.7%; 114/1474) hadn’t tried to access the NHS healthcare due to fear of arrest. This barrier was more common among females (58.8%; 67/114) and those who were undocumented (81.6%; 93/114). In a multivariable analysis, people whose immigration status was ‘undocumented’ were three times more likely not to seek care due to fear of arrest compared to those with permissions to reside (adjusted OR: 3.03; 95% C.I.:1.70-5.40) after adjusting for age, gender and income level. A sensitivity analysis was conducted to examine the effect of missing data and results were consistent with our primary analysis (Table S1, available as *Extended data* ^13^). 

## Discussion

Our study describes the characteristics of the population using DOTW services in London and Brighton, providing a unique and timely insight into a highly excluded population and the likely impacts of the increasingly ‘hostile environment’ imposed on some migrant groups in the UK. This UK-based humanitarian health service evaluation demonstrated that the majority of this population were young, non-EU/EEA nationals and living in poverty. Over half had an undocumented immigration status and close to a fifth had claimed asylum. Identifying groups at greatest risk of facing ‘hostile environment’-related barriers to NHS care, we found a tenth of individuals have in fact sought and been denied NHS care and asylum seekers were at highest risk. Furthermore, seven percent of those presenting at DOTW clinics hadn’t tried to access NHS care due to fear of arrest, and this was three times more likely for those with an undocumented immigration status.

The very fact that individuals are seeking healthcare outside of the NHS, at DOTW clinics, is itself evidence of marginalisation. Our findings are significant in highlighting the multiple and likely intersecting characteristics which may contribute to marginalisation and exclusion, such as poverty and precarious immigration status/undocumentation. The results are consistent with a number of recent studies in Europe concerning the characteristics of undocumented migrants and their healthcare needs ^14, 15^. The Médecins du Monde Observatory Report, which included 43,286 individuals, found that more than half of those reporting ‘fear of arrest’ as an obstacle to seeking healthcare, were in the UK ^12^. More recently, health and legal professionals assisting at Grenfell Tower found victims reluctant to go to hospital because of concerns about their immigration status ^16^. Individuals with undocumented immigration status are the express target of the UK government’s ‘hostile immigration policy’. This study found that undocumented individuals were three times more likely to be afraid of arrest upon accessing NHS care. This provides quantitative evidence supporting the assertion that UK hostile environment policy is deterring individuals from seeking healthcare, when access should not be dependent on immigration status or production of documentation ^17^.

Women migrants are particularly vulnerable to these hostile policies since they are more likely to enter the country as dependents or to be employed in ‘unskilled’ professions ^18^. This makes it more likely for them to be or become undocumented ^18^, and subsequently afraid to access NHS care. Secondly, with the introduction of new charging regulations, the inability to pay a debt for receiving care can negatively affect immigration status and therefore act as a deterrent to seeking care. Our findings are consistent with recent findings that charging leads to avoidance of NHS healthcare because of a fear of charges and the humiliation of being refused care ^19^.

Asylum seekers were found in this study to be twice as likely to have sought and been denied NHS care than those with permissions to reside, attending DOTW clinics. Asylum seekers are legally entitled to the same healthcare rights as ordinary UK residents, so our finding demonstrates that ‘hostile environment’ NHS policies are negatively affecting a wider group than their explicit intention. This is reiterated by a recently published paper that highlights the significant and multifactorial issues faced by asylum seekers when trying to access healthcare, including being wrongly denied access due to having no proof of address or because healthcare providers lack an understanding regarding their entitlement ^20^. A recent systematic review of systematic reviews found migrants experience widespread racism, discrimination, stigma and stereotyping by healthcare professionals ^21^. Poor understanding of migrants’ entitlements and complex gatekeeping systems, little understood by clinicians and administrators, also have a part to play ^22^.

Denying access to primary care has well-recognised public health and economic implications ^11, 23^. Migrants in the UK, for example, utilise screening and treatment adherence programmes less than locally born individuals ^24^, whilst others describe barriers to booking and attending routine vaccinations ^25^. Limiting access to primary care increases A&E attendance and hospital costs ^26^. A recent study in Sicily found undocumented migrants had a higher risk of avoidable hospitalisations due to chronic health conditions, compared to those with documentation ^27^. To further understand the economic impact of policies restricting access to healthcare, we refer to a study in Germany which found that per capita healthcare expenditure was 40% higher among those with restricted access to primary care compared to those with regular access ^28^. The failure to provide good access to primary care and the likely costs associated with subsequent avoidable hospital admissions must be seen in the context of a government that has wholly failed to build a convincing economic rationale or to evaluate the public health cost of its hostile environment policies.

This health service evaluation aimed to improve the understanding of demand for and access to DOTW services. As such our analysis included a full year’s-worth of attendees at DOTW’s clinic, leading to a substantial sample size. DOTW clinics have provided a service, independent of the UK Government, for over a decade. It is likely their services are trusted by migrant communities, meaning this study represents those with the greatest need for healthcare who are excluded from NHS services. Furthermore. data was collected after implementation of 2015 NHS charging regulations ^29^ and provides important insights into the context and likely impact of this fundamental change in policy.

The total number of undocumented migrants in the UK is unknown, making it difficult to draw conclusions regarding the extent to which this group’s health needs are being met, or to estimate the extent or direction of possible biases to those accessing DOTW services. For example, though DOTW offer travel expense reimbursement, services may be less attended by those further away. Newer migrants, more fearful or less knowledgeable, may also be less likely to attend. Lack of population denominator also prevents estimation of the differential 'risk' of population sub-groups (e.g. males vs females) of being excluded from NHS care. Our data only covers attendances during 2016. This means we are unable to investigate the effects of significant policy changes in 2017, when updated charging regulations were enforced, or when the information sharing agreement between NHS Digital and the Home Office was formally retracted in 2018. Furthermore, all information is self-reported, which may lead to inaccuracies; those who are more fearful, for example, may not truthfully report their true immigration status, and others might be embarrassed to admit they are too afraid to access NHS services.

The findings from our health service evaluation make visible the multiple, intersecting vulnerabilities of individuals forced to seek care outside of the NHS. Our results underline the public health imperative for the government to urgently withdraw all of its ‘hostile environment’ policies and address their negative health impacts on these vulnerable individuals, and the wider UK population. Further policy changes in 2017 (e.g. the formal data sharing between NHS Digital and the Home Office; and more charging regulations) have likely only increased the barriers to NHS care that these people face. These ‘hostile environment’ government policies represent an ideologically driven and reckless experiment with the health of these vulnerable people without any evidence base and are not subject to any proper ongoing impact assessment. There is a pressing need to better characterise, understand and meet the healthcare needs of this population and for ongoing research into the public health impact of these policies.

## Data availability

### Underlying data

Underlying data cannot be shared owing to Ethical and Security Considerations. As we describe in our analysis, international migrants accessing DOTW humanitarian health services are often doing so because they are scared to access mainstream NHS services, due to data sharing between the NHS and UK Home Office. For this reason, we are unable to share the underlying data used in this analysis. Researchers wishing to use the data should contact Doctors of The World (email: info@doctorsoftheworld.org.uk) who will review applications on a case by case basis.

### Extended data

Open Science Framework: The negative health effects of hostile environment policies on migrants: A cross-sectional service evaluation of humanitarian healthcare provision in the UK. https://doi.org/10.17605/OSF.IO/G96CW ^13^.

This project contains the following extended data:

- Supplementary material - DOTW Data Collection Form - Social.docx (The ‘social form’ questionnaire used in this assessment).
- Table S1. Logistic regression results of risk factors for denial of access to healthcare and fear of arrest when excluding missing data. .pdf

### Reporting guidelines

Open Science Framework: STROBE checklist for ‘The negative health effects of hostile environment policies on migrants: A cross-sectional service evaluation of humanitarian healthcare provision in the UK’. https://doi.org/10.17605/OSF.IO/G96CW ^13^.

Data are available under the terms of the Creative Commons Zero "No rights reserved" data waiver (CC0 1.0 Public domain dedication).
