# Supplementary material for: The negative health effects of hostile environment policies on migrants: A cross-sectional service evaluation of humanitarian healthcare provision in the UK
Source: Wellcome Open Res. 2019 Jul 22;4:109. [Version 1] doi: 10.12688/wellcomeopenres.15358.1 (PMC6733377; doi:10.12688/wellcomeopenres.15358.1)
Supplement: Peer review history

# Peer review: Reviewer response for version 1

**DOI:** 10.21956/wellcomeopenres.16776.r36158

**Published:** 2019-09-10

**Authors:** Ailish Hannigan

**Affiliations:** 1 Graduate Entry Medical School, University of Limerick, Limerick, Ireland

This paper exploits useful existing data to provide a country specific analysis of characteristics and healthcare needs of marginalized groups.

 The cross-sectional design of the study makes it difficult to draw conclusions about the impact of the policies in the UK. Is it reasonable to assume that no undocumented migrants would have been concerned about getting arrested prior to the ‘hostile environment’ policy? A change in the percentage reporting this over time would have provided stronger evidence of ‘increasing barriers’ and while the discussion uses ‘likely’ impacts, the limitations of no data prior to 2016 needs to be acknowledged. The statement that ‘Most notably the highest numbers of responses reporting fear of arrest and denied access to healthcare was in the UK’ provides some useful evidence of the impact of policy but we are not given the detail of how many of the 43, 286 people in the full Medecins du Monde Observatory Report reported fear of arrest as a barrier to accessing care. Was it common and did it occur even in countries without a hostile environment policy?

 Information on how data is collected on barriers to accessing care would have been useful in the text of the paper itself rather than in the supplementary material. It is important to know the person administering the data collection form is assigning the spontaneous responses of the person attending and the person attending receives at most four prompts for this. Do all individuals attending the clinics report at least one barrier to accessing care? How many reported no difficulties?

 It is clear from the results presented that the main barriers are administrative and lack of knowledge on how to access care. The sentence ‘for undocumented migrants, fear of arrest (81.6%, 93/114) was the healthcare access barrier they were at greatest risk of experiencing’ isn’t clear. From Figure 2A, the healthcare access barrier reported by the highest **number **of undocumented was administrative and not fear of arrest so fear of arrest is not the barrier they were at greatest risk of experiencing. I think it would be useful to give the overall percentage reporting fear of arrest first – (7.7%, 114/1474) and then highlight that the **majority of those reporting fear of arrest** were undocumented (81.6%, 93/114). There is a similar issue with the statement about asylum seekers – it is clear again that the highest number of asylum seekers are reporting administrative barriers but that of the very small number (<10) reporting a previous bad experience, the majority of these were asylum seekers. That doesn’t equate to it being the healthcare access barrier they were at greatest risk of experiencing.

 Even though the numbers will be small, were there any patterns in outcomes by nationality (rather than the category EU national yes/no)? Or by number of attendances? What was the strategy for deciding which explanatory variables were included in Table 1 and then which subset were included in the final logistic regression model (Table 2)? Given the full range of data collected in the data collection form, it may have been interesting to explore patterns in outcomes with reason for migration, or length of stay in the UK – both variables which have been found to be important in other studies. 

 Overall this is a useful data source but needs more careful presentation, clarifications and acknowledgment of limitations.

I confirm that I have read this submission and believe that I have an appropriate level of expertise to confirm that it is of an acceptable scientific standard, however I have significant reservations, as outlined above.

# Peer review: Reviewer response for version 1

**DOI:** 10.21956/wellcomeopenres.16776.r36094

**Published:** 2019-09-06

**Authors:** Mark R. D. Johnson

**Affiliations:** 1 Mary Seacole Research Centre/UK Centre for Evidence in Ethnicity Health & Diversity, De Montfort University, Leicester, UK; 2 Coventry University, Coventry, UK

This is a very topical report which will be of great interest to a wider readership beyond the UK: the issues discussed are very current across the EU and indeed, beyond Europe – and speak to political and Public Health concerns widely. The recent European Public Health Conference, for example, gave considerable space to discussing migrant health, and the European Commission has invested significant amounts into research and policy/practice development regarding health care for migrants and refugees – while DoTW (Medecins du Monde) and other similar groups (including MSF, Red Cross etc) are also engaged in delivering health care to similar groups against this background.

 The paper is well placed in the context of the UN and ICESR where Health is defined as a Human Right, and the issues of the relevance to ‘Public Health’ are quite well laid out – e.g. the impact on ‘herd immunity’ by effective exclusion from vaccination programmes. The cost issues for the national health system are also considered briefly – this is an issue which needs more hard data, but has been explored in a European context by PICUM (the programme for Undocumented Migrants). I would have liked a bit more reference to such relevant contextual research, but accept that the space is limited! Perhaps we can develop this via responses and relevant networks? There is also a useful resource in the Migrant Policy study of access to healthcare across national settings - http://www.mipex.eu/health (Declaration of interest - I helped compile this for UK).

 The paper shows very clearly that there are people who officially do have eligibility for health care and being (or perceiving that they are) refused service on the grounds of migrant status – the statistics might have been clearer displayed with proportions broken down by reasons for refusal against Migration status – rather than showing the proportions of each type of status against reasons for non uptake. It is significant that language is NOT the most important barrier to access, or indeed ‘knowledge’ – it might have been useful to have cited some exemplars of ‘administrative barriers’ (a benefit of qualitative research methods!). The link to the experiences of people affected by the Grenfell Tower disaster is significant and affecting. The ramifications of the ‘Windrush Affair’ might have come too late for inclusion, but that is another similar scandal.

 It might (perhaps in a future similar analysis?) have been of interest to have recorded also ‘Faith’/religion and some measure of ‘race’/ethnicity – does ‘perceived difference’ add to the disadvantage or is personal awareness of being a migrant, in one of the at-risk groups, the dominant factor in self-selection for non-treatment and use of/reliance on this DoTW service?

I confirm that I have read this submission and believe that I have an appropriate level of expertise to confirm that it is of an acceptable scientific standard.

# Peer review: Reviewer response for version 1

**DOI:** 10.21956/wellcomeopenres.16776.r36160

**Published:** 2019-08-19

**Authors:** Maria van den Muijsenbergh

**Affiliations:** 1 Department of Primary Care and Community Care, Radboud University Medical Centre, Nijmegen, The Netherlands; 2 Pharos Centre of Expertise on Health Disparities, Utrecht, The Netherlands

I have thoroughly read and reviewed the article on the negative health effects of hostile environment policies. It addresses a very relevant and disturbing subject, the policies of the UK that inadvertently affect the health of vulnerable groups. I would advise to accept the paper with minor revisions.

 The data in the article are clearly structured and processed and provide interesting and convincing support for the authors' claim that current policies hamper access to Healthcare in certain groups, particular undocumented migrants. Straight forward statistical methods were used that in my opinion (but I am not a statistical expert) were adequate.

 At two places in the paper, I think the authors are a bit too strong in their statements.

 Page 3, left column, four lines from the bottom. They state "Attendance to these clinics is evidence of exclusion...". As we know (and the authors further on also mention this) that not only legal and financial barriers nor fear are reasons to visit these clinics, but also migrants' lack of awareness and knowledge on the Healthcare system, I think this statement is too strong.

 At p. 7 , right column. second sub-para, they link the under attendance to screening programmes by migrants to denying access, but we know that in this under attendance, cultural factors as well as organisational problems not being denied access play an important role.

 I have the following queries:

- Data were gathered in Brighton and London, but nowhere I can see the results per city; I wonder if there are no differences between those two cities.
- I missed the information on the provision of interpreter services during the data collection and consultations.
- I missed the information on the educational level of the participants; as we know, health disparities including barriers to access care, due to lower educational/limited health literacy are substantial, I think this infomation would be relevant.
- Page 5, left column last sentence: "For asylum seekers, a previous bad experience...". For me it is not clear if having a bad experience was the most frequent barrier in accessing care for asylum seekers (and not e.g. fear, lack of money etc.) or that "having a bad experience "was mentioned most frequently by asylum seekers, compared to the other subgroups.

 All in all, an important, good paper, worthwhile for indexing.

I confirm that I have read this submission and believe that I have an appropriate level of expertise to confirm that it is of an acceptable scientific standard.
